# Tuberculosis Caused by* Mycobacterium bovis* in a Sheep Flock Colocated with a Tuberculous Dairy Cattle Herd in Central Ethiopia

**DOI:** 10.1155/2019/8315137

**Published:** 2019-03-03

**Authors:** Benti Deresa Gelalcha, Aboma Zewude, Gobena Ameni

**Affiliations:** ^1^Jimma University School of Veterinary Medicine, P.O. Box 307, Jimma, Ethiopia; ^2^Aklilu Lemma Institute of Pathobiology, Addis Ababa University, P.O. Box 1176, Addis Ababa, Ethiopia

## Abstract

*Mycobacterium bovis* (*M. bovis*) has an exceptionally wide host range including sheep. Information on tuberculosis (TB) in sheep is scarce, and there appears to be conflicting opinions about the relative susceptibility of sheep to infection. In Ethiopia, there was no single previous report on tuberculosis in sheep, though mixed farming of cattle and sheep is a common practice. In this study, following the observation of TB-like lesions on sheep died from sheep flock kept in contact with cattle herd, further investigation was conducted on the flock to assess the magnitude of the infection and identify and characterize the causative* M. bovis strain*. An outbreak investigation was carried out on 26 eligible sheep out of 33 sheep found on the farm. Comparative intradermal tuberculin (CIDT) test, postmortem examination,* Mycobacterium* culturing, and spoligotyping were the techniques used in the study. The prevalence of TB in the tested sheep was 15% (4/26). All the sheep that were positive to CIDT had gross lesions suggestive of TB. Three of the positive sheep had extensive and multiple lesions.* M. bovis* was isolated from all four sheep and the strain was identified as spoligotype SBO134. The in-contact dairy cows were screened for TB and 98% (45/46) of the cows tested positive to CIDT. Furthermore, the same strain, SB0134, was also isolated from the two in-contact cows. The isolation of a matching genotype (SB0134) of* M. bovis* from both species sharing a known epidemiologic link strongly suggests that the sheep flock might have acquired the pathogen from the dairy cows. This warrants strict physical separation of the sheep flock from the cattle herd to prevent such interspecies transmission of* M. bovis*.

## 1. Introduction

*M. bovis* causes tuberculosis (TB) primarily in cattle, although it can infect a wide range of animal species as well as human beings. TB in animals, particularly in cattle, has a devastating effect because of its impact animal productivity and public health significance. TB in cattle leads to strong restriction of trade of live animals and their products with substantial economic losses [1]. As such,* M. bovis *is primarily maintained in cattle and is recognized worldwide as an agent responsible for bovine TB [2, 3].

In Ethiopia, the endemic nature of TB in cattle has long been reported [4] and is one of the countries where TB is widespread in both human and cattle populations [5–7]. More recent reports suggest that the animal level prevalence of bovine TB in Ethiopia ranges from 3.4 % to 50% in different production systems [8–11]. Mixed farming of cattle and small ruminants is a common practice in Ethiopia. Free movement of livestock from one region to another and from farm to farm is also common. Thus, this practice facilitates inter- and intraspecies transmission of* M. bovis*. For example, mixing of cattle and sheep increases the risk of transmission of* M. bovis *from cattle to sheep [9].

Information on TB in sheep is scarce and there appear to be mixed opinions about the comparative susceptibility of sheep to infection with* M. bovis,* and little evidence is available on minimum infective doses. For example, previous studies categorized both sheep and goats as being highly susceptible and indicated that a subcutaneous dose of 1 mg of moist bovine bacilli produces a fatal generalized disease [12], while other authors [3], on the other hand, stated that sheep and horses show a high degree of natural resistance to TB.

Further study indicated that, under circumstances where exposure to infection is high, there is no doubt that sheep can become infected and display lesion morphology and distribution similar to those which occur in cattle [13]. In Ethiopia, the widespread occurrence of bovine tuberculosis has been reported in cattle. Mixed farming of cattle and sheep is commonly practiced in the country posing a high risk of interspecies transmission of pathogen. The aim of the present study was to investigate bovine TB in a flock of sheep kept with a dairy herd with a known history of TB [8–10].

## 2. Materials and Methods

### 2.1. Study Animals and Their Management

The study's sheep were local breeds and they were kept on Government-owned dairy farm with a known history of TB. The farm was located in East Shewa zone of Oromia region in Bishoftu town. The farm had 33 sheep (26 sheep are older than 6 months) and 95% of them are female. In 1996, following the observation of TB-suspicious lesions in a dead cow upon postmortem examination, the dairy herd was tested using the comparative intradermal tuberculin (CIDT) test and the prevalence was 90% (270/300) [14]. During early 2000s, the farm was sold to the private company without culling of any the positive animals.

In 2009, the sheep were introduced into the farm, due to the continued decrease in herd size of the dairy cattle. The sheep flock share the same drinking water tank and same grazing units with the dairy cows. In 2012, TB-like lesions were observed following postmortem examination of one of the sheep that died on the farm. Consequently, the dairy herd were tested for TB using the CIDT.

### 2.2. Comparative Intradermal Tuberculin (CIDT) Test

From a total of 33 sheep kept on the farm, 26 sheep (which are older than 6 months) were screened for TB by CIDT test using avian and bovine purified protein derivatives (PPDs) tuberculins (kindly donated by Dr. Stefan Berg of the Animal and Plant Health Agency, UK). Prior to injection, the right and left sides of the neck of sheep were cleaned and clipped and the site was marked. The thickness of the skin fold was measured with skin calipers and recorded prior to injection. Then, aliquots of 0.1ml (2500IU) avian PPD (Lelystad, Netherlands,) were injected intradermally into a clean clipped area of skin in the middle of the left side of the neck of a sheep, while aliquots of 0.1ml (2000IU) bovine PPD (Lelystad, Netherlands) were injected into a clean clipped skin of the middle neck on the right side. A proper intradermal injection of PPD was ensured by palpating a small pea-like swelling at each site of injection. The skin fold thickness from each injection site was remeasured 72 hours after injection and any difference in absolute skin fold thickness was assessed and interpreted following standard interpretation of the World Organization for Animal Health [15]. The skin reaction in the sheep was considered to be positive if the differential increase in skin thickness at bovine PPD injection site was 4 mm greater than the reaction shown at the site of the avian injection. Similarly, the dairy cattle herd which was in contact with the sheep was tested using CIDT and the result was interpreted as described above.

### 2.3. Postmortem Examination

All positive sheep and two positive dairy cows were slaughtered and subjected to detailed postmortem examination. Postmortem examination involved visual inspection and palpation of lymph nodes and organs such as lungs, liver, and other viscera. Suspicious lesions were further incised and inspected following the procedure described earlier [16]. Tissues with lesions suggestive of TB were collected for bacteriological culture and further identification using spoligotyping.

### 2.4. Mycobacterium Culturing

Specimen processing and culturing for isolation of mycobacteria were carried out at the Aklilu Lemma Institute of Pathobiology (ALIPB) following the protocols of World Organization for Animal Health [17]. Briefly, tissues specimens were sectioned into thin pieces and homogenized with a mortar and pestle. A 4% NaOH was added to the homogenate; and the mixture was centrifuged at 402 g for 15 minutes. The supernatant was discarded and the sediment was neutralized by 1% (0.1N) HCl using phenol red as indicator. Neutralization was considered to be achieved when the color of the solution was changed from purple to orange/yellow.

Thereafter, 0.1ml of the suspension was inoculated onto a duplicate set of Löwenstein-Jensen (LJ) slants: one supplemented with 0.4% sodium pyruvate (LJ pyruvate) and the other with glycerol (standard LJ). Cultures were incubated without CO_2_ at 37°C for at least eight weeks with weekly observation for growth. The culture media were tightly closed to avoid desiccation. Initial identification of mycobacterial species was based on the rate of growth and colony morphology and confirmed by detection of acid-fast bacilli (AFB) by Ziehl–Neelsen staining [18]. Positive cultures were heat-killed in water bath at 80°C for 45 minutes and stored at -20°C until the cells were characterized by spoligotyping.

### 2.5. Spoligotyping

Spoligotyping was performed following the procedure described earlier [19] and also as recommended by the spoligotypes kits supplier's instruction (Ocimum Biosolutions Company, IJsselstein, Netherlands). Briefly, a total volume of 25 *μ*l of reaction mixture consisting of 12.5 *μ*l of HotStarTaq master mix (Qiagen), 2 *μ*l of each of the two primers (20 p mol each), 5 *μ*l suspensions of heat-killed cells, and 3.5 *μ*l sterile water (Qiagen) was used. The mixture was heated for 15 minutes at 96°C and then subjected to 30 cycles of 1 minute at 96°C, 1 minute at 55°C, and 30 seconds at 72°C and a final extension at 72°C for 10 minutes.

The amplified products were hybridized to a set of 43 immobilized spacer oligonucleotides for 1h at 60°C. After hybridization, the membrane was washed twice for 10 minutes in 2x SSPE (1x SSPE is 0.18 M NaCl, 10 *μ*M NaH2PO4, and 1 *μ*M EDTA (pH 7.7)-0.5% sodium dodecyl sulfate at 60°C and then incubated in 1:4000 diluted streptavidin peroxidase (Boehringer) for 45 to 60 minutes at 42°C. The membrane was washed twice for 10 minutes in 2x SSPE-0.5% sodium dodecyl sulfates at 42°C and rinsed with 2x SSPE for 5 minutes at room temperature. Hybridizing DNA (presence or absence of the unique spacers) was detected by the enhanced chemiluminescence method (Amersham) and by exposure to X-ray film (Hyperfilm ECL, Amersham) which detects light signals and thereby produces a pattern that allows for typing of isolates as specified by the manufacturer.

## 3. Results 

### 3.1. The Skin Test Findings

**Figure 1:**
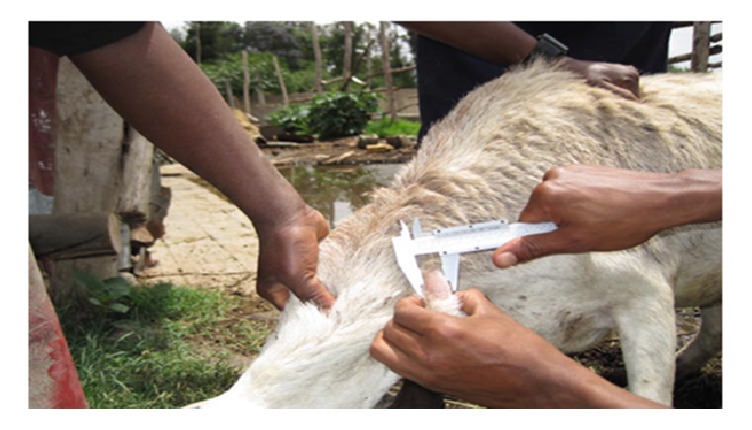
Increased thickness of skin fold of the middle neck of a sheep for comparative intradermal tuberculin test. Skin was swollen at bovine PPD injection site and the sheep was positive for tuberculosis.

The prevalence of skin test rectors in the study sheep flock was 15% (4/26). In all the positive sheep, the differential increase in skin thickness at bovine PPD injection site was greater than 12 mm, suggesting a severe delayed hypersensitivity reaction (Figure 1). The dairy herd was severely affected, with 45 of the 46 dairy cows (98%) having tuberculin reactors.

### 3.2. Postmortem Examination Findings in Sheep

**Figure 2:**
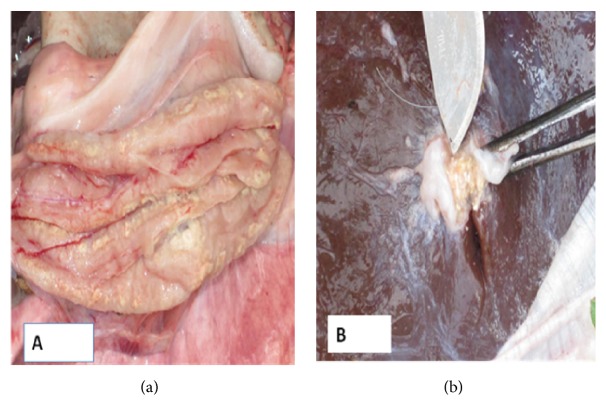
Thick purulent to caseous and calcified tuberculous lesions in the caudal mediastinal lymph node and in the hepatic lymph node observed in two different CIDT reactor sheep. (a) Caudal mediastinal lymph node was incised longitudinally to expose lesions. (b) Incision of hepatic lymph nodes exposed partially calcified TB lesion.

The four sheep that reacted to CIDT test were slaughtered and examined for TB lesions. Gross lesions consistent with mycobacteria infection were detected in different body parts. In three of the infected sheep, lesions ranging from multiple encapsulated, calcified gritty gray nodules to extensive, soft, caseous tissue in the thoracic and abdominal cavities (Figure 2) were observed. Typical TB lesion was also detected in retropharyngeal and submandibular lymph nodes. Lesions in the lungs and liver were also consistent with infection with mycobacteria. In one of the sheep, the lesion was restricted to the mesenteric lymph node. In addition, all of the four sheep were old and have poor body condition and infested with lungworm. Two of the positive cows were slaughtered and thick cream yellowish to crumbly cheese disseminated tuberculous lesions were observed in different body parts of both cows.

### 3.3. Mycobacteriological Examination Result

**Figure 3:**
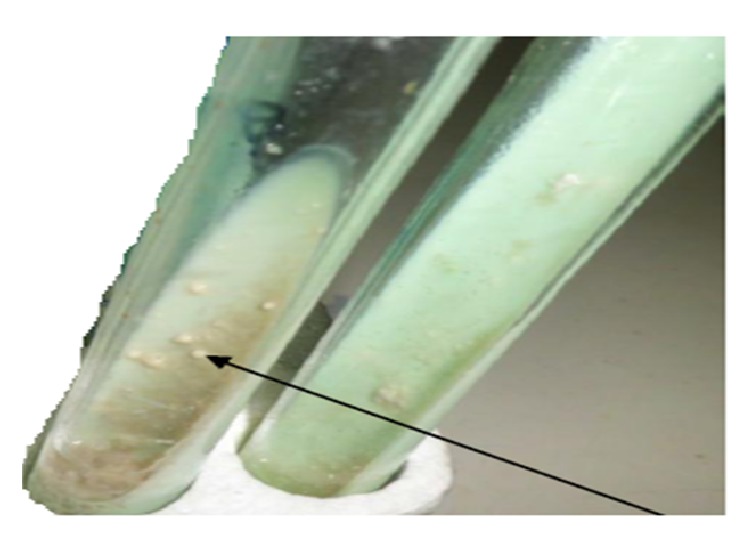
Growth and morphology of* Mycobacterium* colony isolated from tissue samples of tuberculin positive sheep.

Mycobacterial growth was observed on solid Löwenstein-Jensen slopes (Figure 3). When growth was visible, smears were prepared and stained by Ziehl–Neelsen technique and bacterial smears were positive to acid fast test.

### 3.4. Strain Typing

**Figure 4:**
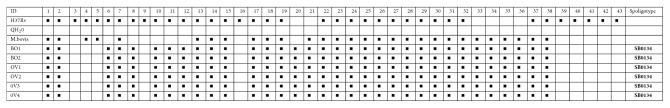
Spoligotype patterns of* M. bovis *isolates from cattle and sheep. The first two samples (BO1 and BO2) were isolates obtained from bovine species (cows) in contact with sheep, while the rest (OV1, OV2, OV3, and OV4) were obtained from the four sheep that were positive to CIDT test and postmortem examination. Positive controls: H37Rv=*M. tuberculosis* H37Rv;* M. bovis 2122/97, *Negative control: Qiagen water (QH_2_O). Both positive control strains yielded standard patterns of spacer arrangements. This is signified by absence of spacers 20-21 and 33-36 and presence of spacers 39-43 for* M. tuberculosis* H37Rv; and absence of spacers 3, 9, 16 and 39-43 for* M. bovis* BCG.

Strain typing of the isolates using spoligotyping confirmed that all of the ovine and bovine isolates were* M. bovis *and identified them all as belonging to spoligotype SB0134 (Figure 4) (URL:http://www.mbovis.org).

## 4. Discussion 

Tuberculosis has been considered to be rare in sheep [20] and published reports tend to describe individual cases rather than outbreaks. Sheep can be infected with* M. bovis *and act as spillover hosts. Infection in a spillover host, as described in the current study, may thus suggest the presence of high levels of* M. bovis* in the environment [21].

The present study reports the first confirmed cases of a TB in sheep flock kept in the same premise with a cattle herd with a known history of tuberculosis in Ethiopia. In this study, fifteen percent of the sheep flock reacted to CIDT test. Similar prevalence was reported earlier in New Zealand in a flock of sheep which was grazing with a cattle herd infected with TB [22]. CIDT test-positive sheep were further confirmed by detection of gross pathology in different tissues and* M. bovis* was isolated from the lesions. Previous studies have documented that sheep do not seem to maintain the infection within their own populations in the absence of continuous acquisition of infection from infected in-contact maintenance hosts [21], suggesting the importance of the presence of other primary hosts that could act as a potential source of infection.

Spoligotyping identified all the bovine and ovine isolates in this study as* M. bovis* SB0134. Absence of spoligotyping spacers 3, 9, 16, and 39–43 is a presumptive indicator of* M. bovis *[19]. This strain (SB0134) has been recently isolated from cattle slaughtered at Addis Ababa and Gondar Abattoirs, northern part of Ethiopia [10, 11, 23]. Moreover, this strain was first reported from Europe [24–26]. It has also been reported from South Africa [27]. It is also one of the most common strains in Mali [28]. It is speculated that this strain could have been introduced into Ethiopia along with imported dairy cows during the start of dairy development operations.

In the present study, gross lesions of tuberculosis were present in all the four tuberculin-reacting sheep. Gross lesions observed in sheep were similar to those described earlier in the same species by other researchers [29, 30]. There appears to be agreement that lesions are mostly caseous and well encapsulated [22]. In the present study, three of the infected sheep showed extensive and multiple lesions (in both thoracic and abdominal cavities), making the determination of the route of entry difficult to ascertain. In one of the sheep, the lesion was restricted only to the mesenteric lymph node, suggesting ingestion as the probable route of infection. It was indicated that the route of transmission of* M. bovis *within the same species or between different species can be deduced by the pattern of lesions observed in slaughtered animals [31]. Animals with lesions restricted to the thoracic cavity are presumed to have been infected by the inhalation of aerosols, while those with lesions restricted to mesenteric lymph nodes are thought to have acquired the infection by ingestion [32].

The prevalence of TB in dairy cattle herd was 98% (45/46). The very high prevalence of the infection in the herd could be due to lack of disease management (isolation and culling of positive animals). Postmortem examination was conducted on two progressively ill cows and typical TB gross lesions were observed in both cows. The lesions were further confirmed with isolation of the same strain (SB0134) of* M. bovis *from cattle. The sheep had been housed in a building used to house cattle, almost all of which were then shown to be infected. The most likely explanation is that the sheep might have acquired infection from the cattle herd because they are the primary hosts for the pathogen and due to previous history of exposure to the infection.

The sheep share the same grazing land, watering troughs, and adjacent premises together with a dairy herd.* M. bovis* may establish itself in the lymph nodes of the digestive tract and splashing of the contaminated water could also provide a means of entry of bacilli into the respiratory tract of sheep. The very small numbers of sheep found to be infected with* M. bovis* in this study are not consistent with the proposition that sheep are highly susceptible to this infection [12, 33]. If sheep were equally susceptible to infection as cattle herds, then similar prevalence of infection would be expected in the sheep flock. This observation suggests that sheep can become infected only when the level of challenge is relatively high.

According to some authors [30], TB is rare in sheep because of the lack of opportunity for infection and not because sheep are innately resistant to TB. According to this author, the low incidence of TB in sheep is a consequence of management and behavioral factors, which tend to reduce their exposure to this pathogen. Another report [28] indicated that when sheep are exposed to high infection, there is no doubt that they become infected, as can be substantiated by the findings of the present study.

## 5. Conclusion 

The present study showed the occurrence of severe gross TB lesions in sheep kept closely with cattle herd with high rate of TB infection. There was marked difference in measured prevalence of TB in cattle and sheep. The detection of TB in only four sheep in the flock tends to place sheep as more resistant to the infection than cattle (they require a much higher infective dose than cattle before infection can become established). This suggests that the infection in sheep is only a symptom of infection in other in-contact reservoir species and does not easily pass from sheep to sheep. However, the presence of lesions in the sheep respiratory tract has an implication that sheep may act as a source of infection for other animals including human. The isolation of the same strain (SB0134) of* M. bovis* from cattle and sheep sharing a known epidemiologic link indicates that the infection could have spilled over from cattle to the in-contact sheep. This warrants strict physical separation of sheep flock from cattle herd to prevent such interspecies transmission of* M. bovis* and reduce its zoonotic risk.

## Data Availability

By virtue of its nature, this manuscript has very limited data except the raw data on tuberculin skin test result of the 26 sheep. In addition, there are also some photos showing a sheep flock and a dairy herd grazing on the same plot of grazing land. The authors can provide additional photos and the raw data if the need arises; otherwise, all important photos are included in the submitted manuscript.

## List of Abbreviations

ALIPB: Aklilu Lemma Institute of Pathobiology
AFB: Acid fast bacilli
BTB: Bovine tuberculosis
CIDT: Comparative intradermal tuberculin test
DNA: Deoxyribonucleic acid
PPD: Purified protein derivative
TB: Tuberculosis.
